# Effects of coconut meal inclusion on growth performance, nutrient utilization, carcass characteristics, and meat quality in Awassi Lambs

**DOI:** 10.14202/vetworld.2025.1411-1420

**Published:** 2025-06-06

**Authors:** Ja’far Al-Khaza’leh, Belal Obeidat

**Affiliations:** 1 Department of Nutrition and Food Processing, Faculty of Agricultural Technology, Al-Balqa Applied University, P.O. Box. 19117, Al-Salt, Jordan; 2 Department of Animal Production, Faculty of Agriculture, Jordan University of Science and Technology, Irbid, 22110, Jordan

**Keywords:** Awassi lambs, carcass characteristics, coconut meal, cost efficiency, meat quality, nutrient digestibility

## Abstract

**Background and Aim:**

Feed cost constitutes a major constraint in small ruminant production systems in Jordan. The search for alternative, cost-effective feed ingredients has prompted interest in coconut meal (COC), a by-product of coconut oil extraction. This study evaluated the effects of including 10% COC (COC10) in the diet on the growth performance, nutrient utilization, carcass characteristics, and meat quality of growing Awassi lambs.

**Materials and Methods:**

Twenty-four male Awassi lambs (17.2 ± 0.63 kg) were randomly assigned to two dietary treatments: A control diet without COC (CON) and a test diet with 100 g/kg dry matter (DM) COC (COC10). The feeding trial lasted 70 days, comprising a 7-day adaptation and a 63-day experimental period. Feed intake and growth performance were monitored throughout. On day 49, five lambs per group were used to assess nutrient digestibility and nitrogen balance using total fecal and urine collection in metabolic cages. On day 64, lambs were slaughtered to evaluate carcass characteristics and meat quality. Blood samples were collected for serum biochemical analysis.

**Results:**

The inclusion of COC10 significantly increased acid detergent fiber and ether extract intake (p ≤ 0.05), with no adverse effects on DM intake, weight gain, feed efficiency, or nutrient digestibility (p > 0.05). Economic analysis revealed a 16% reduction in production cost per kg of weight gain in the COC10 group (p = 0.05). No significant effects were observed on carcass traits, meat quality parameters, or blood biochemical profiles.

**Conclusion:**

Incorporating COC10 into the diet of Awassi lambs had no detrimental impact on growth, carcass characteristics, or health status, while improving economic efficiency. COC is a viable alternative protein and energy source in lamb diets. Further research is warranted to determine optimal inclusion rates under varying production systems.

## INTRODUCTION

The rising demand for cost-effective and sustainable feed alternatives has heightened interest in utilizing non-conventional resources, particularly in regions affected by feed scarcity and volatile global prices of conventional feedstuffs, which have hind-ered livestock sector development. In Jordan, feed expen-ditures represent nearly 75% of the total variable costs on small ruminant farms [1]. Thus, integrating alternative feed ingredients, including agro-industrial by-products, presents a practical approach to mitigating these challenges and enhancing economic returns.

The coconut palm (*Cocos nucifera*) is widely cultivated across tropical and subtropical areas for various purposes [2, 3]. The Asia-Pacific region alone contributes approximately 88.7% of global coconut production [4]. Based on the latest data from the Food and Agriculture Organization [4], coconut plantations spanning 11.05 million hectares produced 62.41 million metric tons (MMT) of coconuts in 2022. Forecasts suggest that global production will rise to 65.4 MMT by 2026 [5].

Coconut meal (COC), or copra meal, is a major by-product generated during coconut oil extraction, with increasing output driven by global demand for coconut-derived products [6]. According to the United States Department of Agriculture [7], global copra meal production was estimated at 1.92 MMT.

COC has emerged as a prominent component in ruminant feeding programs. It accounts for roughly 28%–37% of the original coconut copra mass [8, 9]. As an economical feedstuff, COC delivers both energy and protein at a reduced cost [2]. Lee-Rangel *et al*. [10] reported that COC provides an energy yield of 4.7 Mcal/kg of dry matter (DM), highlighting its utility in ruminant diets. Nutritionally, COC contains approximately 22.94% crude protein (CP), 0.50% lysine, 0.36% methionine, 13.04% crude fiber (CF), 9.00% ether extract (EE), and 6.88% total ash on a DM basis [2]. However, its nutrient profile can vary depending on coconut maturity, drying practices, storage methods [2, 11], and the oil extraction technique employed, whether mechanical or solvent-based [12].

Numerous studies have explored the impact of COC on animal performance. Paengkoum [13] found that substituting up to 50% of soybean meal with COC in goat diets had no negative impact on feed intake, digestibility, or body weight (BW) gain. Lee-Rangel *et al*. [10] reported comparable growth rates in lambs consuming diets with 50, 100, or 150 g/kg DM of copra meal relative to a control diet, although all levels of inclusion increased feed conversion ratios. Conversely, Sundu *et al*. [14] observed that incorporating 0% to 50% coconut by-products in broiler rations led to marked reductions in feed intake, weight gain, feed efficiency, DM digestibility, and metabolizable energy. Dairo and Fasuyi [15], however, demonstrated that fermented COC protein could replace up to half the soybean meal in laying hen diets without significantly impairing performance.

Additional studies have examined COC’s influence on growth and carcass parameters. O’Doherty and McKeon [16] observed no significant performance differences in pigs during the grower-finisher phase when 20% of barley was replaced with COC in a least-cost formulation. Similarly, Hammond and Wildeus [17] found that COC supplementation in growing lambs enhanced dressing percentage and increased slaughter weight. Furthermore, COC offers a cost-effective alternative to more expensive cereal grains in animal nutrition. Research by Obeidat *et al*. [18] demonstrated that COC supplementation in nursing ewes lowered milk production costs while enhancing both milk output and preweaning growth in lambs.

Despite the increasing global interest in incorporating agro-industrial by-products such as COC into ruminant diets, empirical data on the efficacy and safety of COC inclusion in small ruminant production systems remain limited, particularly in the context of the Middle East. Most existing studies have foc-used on tropical and subtropical regions outside the Mediterranean zone, with limited transferability due to differences in feeding systems, animal breeds, and environmental conditions. In addition, while the use of COC has been investigated in various livestock species, studies evaluating its impact on nutrient utilization, carcass traits, and meat quality in Awassi lambs – a predominant fat-tailed breed raised under semi-intensive systems in Jordan – are scarce. Moreover, existing research has often focused on growth performance outcomes without concurrently assessing digestibility, nitrogen balance, or comprehensive economic efficiency indicators. This lack of integrated assessment restricts the practical application of findings for formulating cost-effective and nutritionally balanced rations for Awassi lambs under local production settings.

This study aimed to evaluate the effects of dietary inclusion of 10% COC (COC10), as a partial replacement for conventional protein and energy sources (soybean meal and barley grain), on growth performance, feed intake, nutrient digestibility, nitrogen balance, carcass characteristics, meat quality, and serum biochemical parameters in growing Awassi lambs raised under Jordanian production conditions. A further objective was to assess the cost-effectiveness of COC-based diets to determine their viability as sustainable and economical alternatives. The findings from this study are expected to contribute to the development of region-specific feeding strategies that enhance livestock productivity while reducing input costs and dependence on imported feedstuffs.

## MATERIALS AND METHODS

### Ethical approval

Before the commencement of the study, all experimental procedures were reviewed and approved by the Institutional Animal Care and Use Committee of Jordan University of Science and Technology (JUST) (Approval number: 16/04/12/39AB).

### Study period and location

The study was conducted from January 2024 to March 2024 at the Agricultural Research and Training Unit, Faculty of Agriculture, Jordan University of Science and Technology.

### Animals, housing, and management

Before the start of the experiment, Awassi lambs were weighed, assessed for health status, and treated against internal parasites. Lambs were 2.5–3 months old and had similar initial BWs, averaging 17.2 ± 0.63 kg. The lambs were individually housed in pens (1.5 × 0.75 m) with concrete flooring. Each pen was equipped with a plastic feeder and a 10 L waterer.

### Experimental design and diets

#### Experimental groups

Out of 50 lambs born at the Animal Research Farm at JUST, 24 male lambs were randomly selected and assigned to two dietary groups of equal size (n = 12). The dietary treatments differed in their levels of COC inclusion. Lambs in the control group (CON) were fed 0% COC, while lambs in the treatment group (COC10) were fed COC10 on a DM basis by partially substituting barley grain and soybean meal.

The chemical compositions of the CON and COC10 diets were 91.3% and 91.7% DM, 15.9% and 15.8% CP, 31.8% and 33.2% neutral detergent fiber (NDF), 11.9% and 13.6% acid detergent fiber (ADF), and 0.9% and 2.0% EE, respectively. Both treatment diets were formulated to contain 15.9% CP on a DM basis. The diets were formulated to meet the nutritional requirements of growing Awassi lambs, as outlined by NRC recommendations [19] (Table 1). Feed was offered twice daily at approximately 08:00 and 15:00 h. Throughout the experimental period, lambs had *ad libitum* access to water and feed, with feed amounts adjusted daily to 110% of the previous day’s intake.

**Table 1:** Ingredients and chemical composition of diets-containing coconut meal (COC) fed to Awassi lambs.

Item	Diet^a^		

	CON	COC10	COC
Ingredients (% DM)			
Barley grain, whole	49.5	41.0	
Soybean meal, 440 g/kg CP (solvent)	18.5	17.0	
Coconut meal	0	10.0	
Wheat straw	22.0	22.0	
Alfalfa hay	8.0	8.0	
Salt	0.9	0.9	
Limestone	1.0	1.0	
Vitamin-mineral premix^b^	0.1	0.1	
Feed cost per ton (US$)^c^	428	390	
Nutrients (% DM)			
DM	91.3	91.7	93.6
CP	15.9	15.8	18.4
NDF	31.8	33.2	35.6
ADF	11.9	13.6	21.9
EE	0.9	2.0	12.2

a Diets=Control diet (CON) or 10% COC (COC10) of dietary dry matter (DM).

b Composition per kg (vitamin A, 600,000 IU; vitamin D3, 200,000 IU; vitamin E, 75 mg, vitamin K3, 200 mg; vitamin B1, 100 mg; vitamin B5, 500 mg; lysine 0.5%; DL-methionine, 0.15%; manganese oxide, 4000 mg; ferrous sulfate, 15,000 mg; zinc oxide, 7000; magnesium oxide, 4000 mg; potassium iodide, 80 mg; sodium selenite, 150 mg; copper sulfate, 100 mg; cobalt phosphate, 50 mg, and dicalcium phosphate, 10,000 mg.

c Calculated based on the prices of diet ingredients for 2024. DM=Dry matter, CP=Crude protein, NDF=Neutral detergent fiber, ADF=Acid detergent fiber, EE=Ether extract

### Diet preparation and costing

The COC was sourced from Green Fields Oil Factory, Amman, Jordan. Before being incorporated into the diet, COC was sun-dried and ground. Local market prices were used to calculate the cost of each diet component. Additional expenses, including labor, electricity, and water, were also included in the cost calculations. Experimental diets were prepared biweekly, and representative samples were collected during each preparation to determine chemical composition.

### Experimental timeline and trials

**Figure 1:**
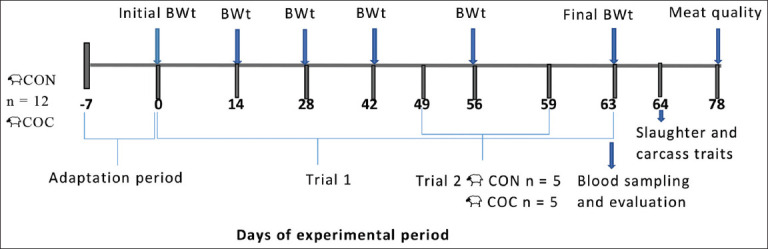
Experimental design.

Figure 1 illustrates the experimental design employed and the measurements taken during the experiment. The experimental period lasted 70 days. The first 7 days (adaptation phase) were used to acclimate the lambs to the diets and pens, while the remaining 63 days (experimental phase) were dedicated to data collection.

In Trial 1, each lamb was weighed on days 1, 14, 28, 42, 56, and 63 of the experimental phase. On day 63, blood samples were collected from each lamb, and on day 64, all lambs were slaughtered. Meat quality was then assessed 2 weeks later (day 78).

In Trial 2, on day 49 of Trial 1, five lambs from each dietary group were randomly selected to assess nutrient digestibility and nitrogen (N) balance. Lambs were adapted to metabolic cages for 5 days, followed by a 5-day data collection period.

### Sample collection and measurements

#### Feed intake and growth performance

Throughout the study period, daily feed consumption was recorded. Lambs were weighed before morning feeding to minimize variation. BW data were used to calculate average daily gain (ADG), total weight gain, and feed efficiency. Feed efficiency was calculated as DM intake (DMI) divided by weight gain.

Diet and refusal samples were collected daily, ground (Brabender OHG Duisburg, Kulturstrasse 51-55, Type 880845, Nr 958084, Germany), and stored for chemical composition analysis. Ground samples were analyzed in duplicate for DM, CP (using the Kjeldahl method), and EE (using the Soxtec method) following Association of Official Analytical Chemists (AOAC) methods [20]. NDF and ADF were determined according to Van Soest *et al*. [21] using an ANKOM2000 fiber analyzer (ANKOM Technology Corporation, Fairport, NY, USA).

#### Digestibility and nitrogen balance

On day 49, five lambs from each dietary group were randomly selected and moved to metabolic crates (1.05 × 0.80 m) for the determination of nutrient digestibility and nitrogen balance. A 5-day adaptation period was followed by 5 days of data collection, during which feed intake, refusals, fecal output, and urinary output were recorded. Subsequently, 5% of urine and 10% of feces were sampled and stored at −20°C for later analysis of DM, CP, NDF, ADF, and EE. Nitrogen content in urine samples was determined using the Kjeldahl method.

#### Blood sampling and biochemical analysis

At the conclusion of the study, blood samples were collected in duplicate from the jugular vein at 08:00 h, before morning feeding, using vacutainer tubes. Blood samples were allowed to clot for 1 h, then centrifuged at 3,000 rpm for 15 min. Serum was separated immediately and stored at −20°C for analysis. Serum concentrations of glucose, urea nitrogen (urea N), alanine aminotransferase (ALT), aspartate aminotransferase (AST), alkaline phosphatase (ALP), total protein, and albumin were measured using a spectrophotometer (Jenway 6105 UV/Vis, Model 6105, Jenway LTD, Felsted, Dunmow, Essex, UK) following the manufacturer’s protocols.

### Slaughter procedure and carcass evaluation

At the end of the study, all lambs were slaughtered at the Animal Farm facilities within the Agricultural Training and Research Unit at JUST. After an 18-h fas-ting period, trained personnel conducted slaughter at 09:00 hours. Fasting live weight and hot carcass weight were recorded immediately before and after slaughter. Non-carcass edible parts (lungs, trachea, heart, liver, spleen, kidneys, renal fat, mesenteric fat, and testes) were removed and weighed.

Carcasses were chilled at 4°C for 24 h before determining dressing percentage, calculated as cold carcass weight divided by fasted live weight. Linear measurements were taken on carcasses and the longissimus muscle, including tissue depth (GR), rib fat depth (J), eye muscle width (A), eye muscle depth (B), eye muscle area, and fat depth (C). Carcasses were divided into four primal cuts: shoulder, rack, loin, and leg. The longissimus muscle from the loin was vacuum-packed and stored at −20°C for 2 weeks for meat quality analysis.

### Meat quality assessment

Meat quality parameters were assessed on longissimus muscle samples after thawing overnight at 4°C. Measurements included shear force, color (CIE Lab*), water-holding capacity (WHC), pH, and cooking loss (CL). For color evaluation, 15 mm thick slices were placed on polystyrene trays, covered with porous film, and allowed to oxygenate at 4°C for 2 h.

For CL determination, 25 mm thick slices were weighed, sealed in plastic bags, and cooked in a water bath at 75°C for 90 min. Post-cooking weight was recorded to calculate water loss. Slices were stored at 4°C overnight before shear force testing. Six meat cores (1 cm diameter) were cut perpendicular to the muscle fibers and sheared using a Warner-Bratzler device (Salter Model 235, G-R Manufacturing Co., Manhattan, KS, USA) [22].

pH was measured using a waterproof pH spear (Model 35634-40, Eurotech Instruments, Shah Alam, Malaysia) after homogenizing 2 g of fresh meat in 10 mL of neutralized 5 mm iodoacetate solution.

WHC was assessed using Grau and Hamm’s method [23]. Five grams of raw beef were minced, placed between quartz plates and filter papers, and compressed with a 2.5 kg weight for 5 min. WHC (%) was calculated as:

WHC% = ([Initial weight − Final weight]/Initial weight) × 100

### Statistical analysis

All data were analyzed using the PROC MIXED procedure in SAS (Version 8.1, SAS Institute Inc., Cary, NC, USA). The treatment diet was used as a fixed effect, and lamb was treated as a random effect. Assumptions of residual normality and homoscedasticity were tested before applying the model. Least-squares means were compared using pairwise t-tests when fixed effects were significant (p ≤ 0.05). The final statistical model used for analysis is detailed below:

Y_ij_= μ + D_i_+L_j_ +ε_ij_

Here, Y_ij_ is the response variable, μ is the overall mean, D_i_ is the fixed effect of diet treatment, L_j_ is the random effect of the lamb, and ε_ij_ is the random error.

## RESULTS

### Chemical composition of COC and experimental diets

The chemical composition of COC and the experimental diets is presented in Table 1. The analysis of COC revealed higher concentrations of CP (18.4 g/kg), NDF (35.6 g/kg), ADF (21.9 g/kg), and EE (12.2 g/kg) compared with the experimental diets. The inclusion of COC10 did not significantly alter the DM or CP content of the diets. However, COC inclusion marginally increased the levels of EE, ADF, and NDF. Furthermore, the COC10 diet was associated with a reduction in feed cost compared with the CON diet.

### Nutrient intake and growth performance

The effects of dietary COC inclusion on nutrient intake and growth performance in Awassi lambs are shown in Table 2. There were no significant differences (p > 0.05) in the overall nutrient intake between the CON and COC10 groups, except for ADF and EE. Lambs fed the COC10 diet had significantly higher intakes of ADF (166 g/day) and EE (25 g/day) compared to those fed the CON diet (145 g/day ADF and 11 g/day EE) (p ≤ 0.05). A non-significant trend toward increased NDF intake (p = 0.07) was also observed in the COC10 group.

**Table 2:** Effects of coconut meal (COC) supplementation on nutrient intake and growth performance of Awassi lambs.

Item	Diet^a^			

	CON (n=12)	COC10 (n=12)	SEM	p-value
Nutrient intake (g/day)				
DM (g/day)	1221	1208	30.3	0.6102
CP (g/day)	194	194	4.9	0.8998
NDF (g/day)	388	408	9.9	0.0704
ADF (g/day)	145	166	3.7	0.0002
EE (g/day)	11	25	0.4	<0.0001
Initial weight (kg)	17.8	16.6	0.63	0.0965
Final weight (kg)	30.9	30.2	1.15	0.5192
Total gain (kg)	13.2	13.5	1.06	0.7303
Average daily gain (g/day)	209	215	16.8	0.7318
Feed efficiency (DMI: ADG)^b^	6.1	5.7	0.46	0.3151
Cost of gain ($US/kg)	2.63	2.21	0.193	0.0501

a Diets=Control diet (CON) or 10% COC (COC10) of dietary dry matter (DM).

b DMI: ADG=Dry matter intake: Average daily gain. SEM=Standard error of the mean, DM=Dry matter, CP=Crude protein, NDF=Neutral detergent fiber, ADF=Acid detergent fiber, EE=Ether extract

Growth performance indicators were not significantly affected (p > 0.05) by the inclusion of COC. Feed efficiency, calculated as the ratio of DMI to ADG, remained unchanged between treatments. However, production cost per kilogram of weight gain was significantly reduced by 16% in the COC10 group (2.21 USD/kg) compared to the CON group (2.63 USD/kg) (p = 0.05).

### Nutrient digestibility and nitrogen balance

Table 3 provides data on nutrient digestibility and nitrogen balance. Throughout the experimental period, no significant differences (p > 0.05) were observed between the CON and COC10 groups in any of the measured digestibility parameters or nitrogen balance values.

**Table 3:** Effects of coconut meal (COC) supplementation on digestibility and nitrogen balance in Awassi lambs.

Item	Diet^a^			

	CON (n=5)	COC10 (n=5)	SEM	p-value
Digestibility coefficients				
DM	78.81	79.83	3.205	0.7645
CP	79.81	80.71	4.932	0.8650
NDF	66.47	65.02	2.214	0.5483
ADF	57.23	58.66	3.062	0.6657
EE	83.17	85.54	1.269	0.2566
N balance				
N intake (g/day)	31.45	31.67	0.711	0.7745
N in feces (g/day)	7.74	7.55	0.653	0.7856
N in urine (g/day)	9.21	8.81	1.897	0.8449
N retained (g/day)	14.50	15.31	1.509	0.4186
Retention (g/100 g)	46.01	48.48	4.601	0.4825

a Diets=Control diet (CON) or 10% COC (COC10) of dietary dry matter (DM). SEM=Standard error of the mean, DM=Dry matter, CP=Crude protein, NDF=Neutral detergent fiber, ADF=Acid detergent fiber, EE=Ether extract

### Carcass characteristics

The effects of dietary COC inclusion on carcass traits and loin tissue characteristics are presented in Table 4. No statistically significant differences (p > 0.05) were detected between the two groups for any carcass parameters. However, lambs in the COC10 group showed trends toward lower hot carcass weight (p = 0.08), subcutaneous fat thickness (p = 0.06), and meat-to-bone ratio (p = 0.08) compared with the CON group.

**Table 4:** Effects of feeding coconut meal (COC) on carcass characteristics and loin tissues of Awassi lambs.

Item	Diets^a^			

	CON (n=12)	COC10 (n=12)	SE	p-value
Fasting live weight (kg)	30.74	29.00	1.180	0.1686
Hot carcass weight (kg)	14.73	13.47	0.656	0.0807
Cold carcass weight (kg)	13.66	12.80	0.695	0.2431
Dressing percentage	44.28	44.13	0.809	0.8524
Non-carcass components (kg)^b^	1.26	1.16	0.088	0.3060
Carcass cut weights (kg)^c^	11.51	11.12	0.535	0.4788
Fat tail (kg)	1.24	1.16	0.117	0.4659
Loin				
Intermuscular fat (%)	2.22	2.66	0.324	0.2018
Subcutaneous fat (%)	8.71	6.92	0.865	0.0623
Total fat (%)	10.87	9.58	0.953	0.2018
Total lean (%)	54.52	52.26	1.628	0.1930
Total bone (%)	28.37	31.55	2.555	0.2387
Meat-to-bone ratio	2.09	1.22	0.188	0.0750
Meat: fat ratio	3.70	4.75	0.595	0.1036

a Diets: Control diet (CON) or 10% COC (COC10) of dietary dry matter (DM).

b Non-carcass components (heart, liver, spleen, kidney, and lungs and trachea).

c Carcass cut (shoulder, racks, loins, and legs). SE=Standard error

### Carcass linear measurements

Carcass linear measurement results are summ-arized in Table 5. No significant differences were observed in rib fat depth (p = 0.71), eye muscle width (p = 0.25), eye muscle depth (p = 0.87), or fat depth (p = 0.41) between groups. Nevertheless, lambs receiving the COC10 diet tended to exhibit lower leg fat depth (p = 0.07), tissue depth (p = 0.08), and shoulder fat depth (p = 0.08) compared with those fed the CON diet.

**Table 5:** Effects of coconut meal (COC) on carcass leaner dimensions of Awassi lambs.

Item	Diets^a^			

	CON (n=12)	COC10 (n=12)	SE	p*-*value
Leg fat depth (*L3*) (mm)	2.18	1.60	0.292	0.0698
Tissue depth (*GR*) (mm)	9.38	8.08	0.671	0.0807
Rib fat depth (*J*) (mm)	1.65	1.74	0.237	0.7061
Eye muscle width (*A*) (mm)	49.92	48.45	1.202	0.2485
Eye muscle depth (*B*) (mm)	19.96	20.13	0.946	0.8654
Fat depth (*C*) (mm)	1.57	2.49	1.071	0.4115
Shoulder fat depth (*S2*) (mm)	1.73	1.34	0.206	0.0830

a Diets=Control diet (CON) or 10% COC (COC10) of dietary dry matter (DM), SE=Standard error

### Meat quality attributes

Meat quality parameters are shown in Table 6. The inclusion of COC10 did not significantly affect meat pH (p = 0.11), CL (p = 0.19), WHC (p = 0.40), shear force (p = 0.25), meat whiteness (p = 0.10), or redness (p = 0.27). However, meat from lambs fed the COC10 diet exhibited a tendency toward increased yellowness (p = 0.08) compared to the CON group.

**Table 6:** Effects of feeding coconut meal (COC) on meat quality characteristics of Awassi lambs.

Item	Diets^a^			

	CON (n=12)	COC10 (n=12)	SE	p-value
pH^b^	5.90	5.85	0.0300	0.1072
Cooking loss (g/100 g)	38.51	40.37	1.322	0.1853
Water-holding capacity (g/100 g)	31.75	33.07	1.501	0.3964
Shear force (kg/cm^2^)	6.75	6.07	0.563	0.2516
Color coordinates				
L* (whiteness)	33.84	32.02	1.012	0.0991
a* (redness)	1.76	1.52	0.202	0.2733
b* (yellowness)	18.16	19.70	0.792	0.0789

a Diets=Control diet (CON) or 10% COC (COC10) of dietary dry matter (DM).

b pH measured after thawing. SE=Standard error

### Serum biochemical profile

No significant effects (p > 0.05) of dietary COC inclusion were found on serum biochemical parameters (Table 7). The experimental diets did not influence blood concentrations of urea nitrogen, cholesterol, glucose, high-density lipoprotein (HDL), low-density lipoprotein, triglycerides, creatinine, AST, ALT, and ALP.

**Table 7:** Effects of feeding coconut meal (COC) on blood parameters of Awassi lambs.

Item	Diets^a^			

	CON (n=12)	COC10 (n=12)	SE	p-value
Urea nitrogen (mg/dL)	20.19	21.28	1.194	0.5293
Glucose, mg/dL	54.13	49.04	3.862	0.2145
Cholesterol, mg/dL	45.25	49.20	4.108	0.3569
Triglycerides, mg/dL	21.33	20.17	3.745	0.7615
High-density lipoprotein (mg/dL)	28.21	31.41	1.962	0.1308
Low-density lipoprotein, mg/dL	12.44	12.83	2.966	0.8980
Aspartate aminotransferase (IU/L)	46.92	41.13	4.116	0.1872
Alanine aminotransferase (IU/L)	12.42	9.49	1.685	0.1101
Alkaline phosphatase, IU/L	86.67	80.35	8.303	0.4627
Creatinine, mg/dL	0.67	0.67	0.068	0.9667

a Diets=Control diet (CON) or 10% COC (COC10) of dietary dry matter (DM), SE=Standard error

## DISCUSSION

### Feed cost and composition of COC

A nutritionally balanced diet is essential during the critical growth phase of lambs; however, such diets are often economically burdensome. Consequently, cereal grains in ruminant rations are increasingly being replaced with alternative feed ingredients to reduce costs. The chemical composition of the COC used in this study was consistent with previously reported values [9, 24]. Factors such as coconut variety, maturity at harvest, drying methods, and storage conditions can influence the chemical composition of COC [25]. In addition, the oil extraction process significantly affects its nutrient profile. As noted by Da Silva *et al*. [26], the oil extraction process elevates EE, NDF, and ADF contents in the resulting meal. Similarly, Paengkoum [13] demonstrated that replacing soybean meal with COC increased NDF and ADF concentrations.

In the present study, COC inclusion increased the dietary EE, NDF, and ADF contents, likely due to COC’s inherent high levels of EE and CF [2]. Feed cost-effectiveness was enhanced by approximately 9% in the COC10 diet relative to the control. This finding aligns with previous reports by Sandy *et al*. [27], Siebra *et al*. [28], and Diarra [29], who noted cost reductions with COC use. As feed cost is the largest variable cost and a major constraint in sheep production in Jordan [1], incorporating COC could promote profitability, sustainability, and resilience among smallholder farms.

### Nutrient intake and growth performance

Nutrient intake was not significantly different between groups, except for ADF and EE, which were elevated in the COC10 group due to the higher content of these components in the diet (21.9% and 12.2%, respectively). The unaltered DMI suggests that palatability and metabolic acceptability of the diet were not compromised. The fatty acid profile and moderate inclusion level of COC likely contributed to this outcome.

These findings are in agreement with Jordan *et al*. [30], who reported no effect of copra meal on DMI in beef heifers, and Lee-Rangel *et al*. [10], who observed no changes in DMI in Rambouillet lambs fed 5%, 10%, or 15% COC. Conversely, Aregheore [31] found decreased feed intake with higher COC inclusion in goats, while Obeidat *et al*. [18] and Paengkoum [13] reported increased DMI at 7.5% and up to 50% COC inclusion, respectively.

Other studies by Da Silva *et al*. [26], Asih *et al*. [32], and Bosa *et al*. [33] yielded mixed results. Da Silva *et al*. [26] observed reduced CP, NDF, and ADF intake in Santa Inês lambs, though EE intake increased. Asih *et al*. [32] noted that nutrient intake in Etawah does remained stable with increased COC, and Bosa [33] found protein intake declined with rising COC levels. Differences in outcomes may stem from variations in COC levels, diet composition, animal species, physiological stages, or environmental conditions.

### BW gain and feed efficiency

In the present study, COC10 inclusion did not improve BW gain, which is consistent with Lee-Rangel *et al*. [10]. While diets with higher digestibility are typically associated with improved growth, no such benefit was observed here. Contrasting reports from Aregheore [31], Obeidat *et al*. [18], Asih *et al*. [32], Jordan *et al*. [30], and Duy and Khang [34] demonstrated enhanced weight gain with varying levels of COC. However, Paengkoum [13] reported reduced gains at 75% inclusion.

Feed efficiency was not improved in this study. In contrast, Hammond and Wildeus [17] found a notable increase in feed efficiency in lambs supplemented with COC.

### Nutrient digestibility and nitrogen balance

Digestibility and nitrogen balance were similar between dietary treatments, likely due to comparable DMI (1,221 vs. 1,208 g/day). This suggests that COC inclusion did not impair ruminal microbial activity or nutrient utilization. Despite its high saturated fatty acid content (92.2%), coconut oil’s potential to mitigate methane and redirect energy was not evident here [35].

The digestibility of the COC10 diet (79.85%) is considered high according to Van Soest [36], and the similarity in protein digestibility between COC, corn, and soybean meals is notable. Pereira *et al*. [37] reported that COC protein has low rumen degradability, high bypass capacity, and excellent digestibility. Supporting studies by Da Silva *et al*. [26] and Paengkoum [13] suggest that, except for reduced NDF digestibility, DM, CP, and EE digestibility were not significantly affected. Aregheore [31] and Obeidat *et al*. [18] observed enhanced digestibility with COC inclusion, while Jordan *et al*. [30] reported declines in DM, OM, and CP digestibility.

Nitrogen balance remained unaffected, likely due to similar protein intake and digestibility. Paengkoum [13] and Obeidat *et al*. [18] reported improved N retention with COC inclusion, while Da Silva *et al*. [26] found reduced N intake and retention at 22% COC. Galgal *et al*. [38] also noted increased N intake with copra expeller pellets.

### Carcass traits and meat quality

Carcass characteristics were not significantly affected by COC inclusion. Similar slaughter and carcass weights explain the observed consistency in dressing percentage and carcass cut weights. Jordan *et al*. [30] reported no effect of copra meal on carcass traits in beef heifers. Conversely, Hammond and Wildeus [17] observed improvements in rib-eye area, back fat, and leg conformation. Diarra [29] and O’Doherty and McKeon [16] reported variable results depending on COC level and animal species.

Meat quality traits were also unaffected. This supports previous work by Jang *et al*. [39], who found no negative impact of COC on pork quality. Diet composition, especially fat type, influences carcass fat deposition and meat traits [40, 41]. Bhatt *et al*. [35] similarly found that coconut oil did not alter carcass or meat characteristics in Malpura lambs.

### Blood biochemistry

No significant differences were observed in blood metabolites or liver enzymes between the groups. Muhlisin [42] also reported no changes in blood cholesterol with coconut-meat waste supplementation. While Obeidat *et al*. [18] noted increased cholesterol and HDL levels, Hu *et al*. [43] and Durand *et al*. [44] observed elevated plasma cholesterol level with coconut oil. Overall, these results suggest that COC inclusion is safe and does not impair lamb health.

## CONCLUSION

This study demonstrated that partial replacement of conventional feed ingredients with COC10 in the diets of growing Awassi lambs did not compromise nutrient intake, digestibility, growth performance, carcass traits, meat quality, or blood biochemical parameters. Specifically, the inclusion of COC significantly increased the intake of ADF and EE without affecting DMI or ADG. Although no improvements in feed efficiency or BW gain were observed, COC inclusion reduced feed cost per kilogram of gain by approximately 16%, indicating enhanced cost-effectiveness. Nutrient digestibility and nitrogen balance remained comparable between diets, suggesting no adverse effects on rumen function or nutrient utilization. Moreover, meat quality attributes, including pH, shear force, CL, and color coordinates were unaffected, supporting the safety and palatability of COC-based diets.

The economic advantage of COC incorporation, alongside its nutritional adequacy, highlights its potential as a sustainable, locally available alternative feed resource for small ruminants. This is particularly relevant in feed-deficient regions such as Jordan, where rising grain prices pose economic challenges to smallholder farmers.

The study’s robust experimental design, which included multiple assessments of intake, digestibility, blood biochemistry, carcass traits, and meat quality over a 70-day period, strengthens the reliability of the findings.

This trial was limited to a single inclusion level (10%) and a specific breed (Awassi lambs) under controlled conditions. Broader applicability across different breeds, production systems, and higher COC inclusion levels requires further validation.

Future studies should evaluate the long-term effects of higher inclusion rates of COC on reproductive performance, methane emissions, and rumen microbiota, as well as its interaction with other agro-industrial by-products in multi-component diets.

The findings underscore the nutritional and economic viability of COC as a partial replacement for soybean meal and barley in lamb diets. The strategic utilization of COC can contribute to sustainable livestock production in resource-constrained settings without compromising animal health or product quality.

## DATA AVAILABILITY

The supplementary data can be made available by the corresponding author on request.

## AUTHORS’ CONTRIBUTIONS

JA and BO: Conceptualized and designed the study, methodology, data curation and interpretation, and drafted and revised the manuscript. Both authors have read and agreed to the final version of the manuscript.
